# Developmental regulation of key gluconeogenic molecules in nonhuman primates

**DOI:** 10.14814/phy2.12243

**Published:** 2014-12-18

**Authors:** Lisa L. McGill‐Vargas, Teresa Johnson‐Pais, Marney C. Johnson, Cynthia L. Blanco

**Affiliations:** 1 Department of Pediatrics, Division of Neonatology, University of Texas Health Science Center at San Antonio, San Antonio, Texas, USA; 2 Department of Pediatrics, Division of Child Neurology, Developmental Pediatrics & Genetics, University of Texas Health Science Center at San Antonio, San Antonio, Texas, USA

**Keywords:** Gluconeogenesis, prematurity, glucose regulation, fetal development

## Abstract

Aberrant glucose regulation is common in preterm and full‐term neonates leading to short and long‐term morbidity/mortality; however, glucose metabolism in this population is understudied. The aim of this study was to investigate developmental differences in hepatic gluconeogenic pathways in fetal/newborn baboons. Fifteen fetal baboons were delivered at 125 day (d) gestational age (GA), 140d GA, and 175d GA (term = 185d GA) via cesarean section and sacrificed at birth. Term and healthy adult baboons were used as controls. Protein content and gene expression of key hepatic gluconeogenic molecules were measured: cytosolic and mitochondrial phosphoenolpyruvate carboxykinase (PEPCK‐C and PEPCK‐M), glucose‐6‐phosphatase‐alpha (G6Pase‐*α*), G6Pase‐*β*, fructose‐1,6‐bisphosphatase (FBPase), and forkhead box‐O1 (FOXO1). Protein content of PEPCK‐M increased with advancing gestation in fetal baboons (9.6 fold increase from 125d GA to 175d GA,* P *< 0.001). PEPCK‐C gene expression was consistent with these developmental differences. Phosphorylation of FOXO1 was significantly lower in preterm fetal baboons compared to adults, and gene expression of FOXO1 was lower in all neonates when compared to adults (10% and 62% of adults respectively, *P *< 0.05). The FOXO1 target gene G6Pase expression was higher in preterm animals compared to term animals. No significant differences were found in G6Pase‐*α*, G6Pase‐*β*, FOXO1, and FBPase during fetal development. In conclusion, significant developmental differences are found in hepatic gluconeogenic molecules in fetal and neonatal baboons, which may impact the responses to insulin during the neonatal period. Further studies under insulin‐stimulated conditions are required to understand the physiologic impact of these maturational differences.

## Abstract (precis)

Significant developmental differences were found in several hepatic gluconeogenic molecules. In particular, phosphorylated FOXO1 was significantly reduced in the liver of premature fetal baboons compared to adults and may contribute the increased incidence of hyperglycemia seen in prematurity. In addition, PEPCK increased with advancing gestational age and may play a key role in glucose regulation during the newborn period.

## Introduction

Aberrant glucose regulation is common in the neonatal period, in particular, hyperglycemia of prematurity and hypoglycemia of growth‐restricted fetuses. However, glucose metabolism in this population is understudied and the developmental mechanisms responsible for an effective transition from prenatal to postnatal life remain unknown. Growth‐restricted fetuses and preterm infants are a high‐risk population presenting with significant glucose instability due to limited substrate availability and ineffective glucose disposal (Mersmann 1971; Narkewicz et al. 1993; Mejri et al. 2010). Preterm infants have become the largest clinical population in the neonatal intensive care unit and abnormal glucose metabolism ranging from hypoglycemia to hyperglycemia persists for several weeks and has long‐term effects. For example, neonatal hypoglycemia decreases neurodevelopment and IQ in newborns and hyperglycemia of prematurity is associated with hyperosmolar dehydration, intraventricular hemorrhage, white matter reduction, retinopathy of prematurity, and death (Garg et al. 2003; Hall et al. 2004; Hey 2005; Blanco et al. 2006). Therefore, understanding the mechanisms responsible for glucose control is of greatest importance.

Studies in preterm infants have shown persistence of glucose production despite receiving glucose infusions exceeding glucose turnover rates (Chacko and Sunehag 2010). In addition, in extremely immature infants, gluconeogenesis does not seem to be affected by glucose infusion, plasma glucose, or insulin concentrations (Chacko et al. 2011). Therefore, abnormal expression of key gluconeogenic molecules likely has a major role in the gestational differences seen in endogenous glucose production/suppression, and the neonatal hypo/hyperglycemia seen in prematurity. Endogenous glucose production (EGP) is derived from gluconeogenesis and glycogenolysis. Alterations in the production rates can be caused by variations in gluconeogenesis, glycogenolysis, or both. In preterm infants, gluconeogenesis has been attributed to account for 72% of EGP (Sunehag et al. 1999; Thorn et al. 2013) compared to only 40–60% in adults (Vo et al. 2013). Basal EGP measured in preterm and term infants appears to be the same, but it covers only 40–70% of the requirements in preterms, compared with 60–100% in term infants (Bier et al. 1977; Cowett et al. 1983; Kalhan et al. 1986; Cowett and Wolfe 1991).

In healthy adults, insulin is the primary regulator for gluconeogenesis, which decreases as plasma insulin increases in response to glucose infusions. In addition, gluconeogenesis is regulated primarily through alterations in the expression of three major gluconeogenic enzymes: glucose‐6‐phosphatase (G6Pase), fructose 1,6‐bisphosphatase (FBPase), and phosphoenolpyruvate carboxykinase (PEPCK) (Sekine et al. 2007). Forkhead box O1 (FOXO1) is a key transcription factor for mediating insulin action on gluconeogenesis (Gross et al. 2009). However, the developmental aspects of gluconeogenesis are not well understood as the data available stem from studies in animals with an altered fetal environment during late gestation. Understanding developmental regulation of gluconeogenesis in the fetal/newborn period will optimize treatment and prevention strategies for hypoglycemia and hyperglycemia during the newborn period.

Due to the unethical nature of obtaining liver samples from human infants, an animal model that resembles human development is of utmost importance. Baboons have close (98%) phylogenetic proximity with humans and adult baboons develop insulin resistance/hyperglycemia when obese (Chavez et al. 2008). Hyperglycemia is common in preterm baboons, making them an excellent model to study hepatic gluconeogenesis and the underlying processes responsible for aberrant glucose regulation in the perinatal period (Blanco et al. 2013).

The objective of the present study was to examine developmental differences in hepatic gluconeogenic pathways in fetal/newborn baboons. We hypothesized that gestational differences exist in fetal gluconeogenic proteins and gene expression.

## Materials and Methods

### Animal care

#### Fetal animals

Fifteen fetal baboons were delivered at 125 day (d) gestational age (GA), 140d GA, and 175d GA (term = 185d GA) via cesarean section from healthy, nondiabetic mothers at the University of Texas Health Science Center in San Antonio (UTHSCSA), Texas Biomedical Research Institute (TBRI) in San Antonio, Texas, or at Oklahoma Primate Center, Oklahoma and were sacrificed immediately after birth. Cesarean sections were performed under sterile technique. Female maternal baboons were sedated with ketamine hydrochloride (10 mg/kg) and anesthetized with 2% isoflurane. Postoperative analgesia was provided for the maternal animals using buprenorphine (0.01 mg/kg) twice daily for 2 days. Fetal animals were euthanized immediately after birth with 20 mg/kg of IV pentobarbital, followed by exsanguination. Liver samples were collected and snap frozen.

#### Postnatal animals

Five animals were delivered at term via spontaneous vaginal delivery from healthy, nondiabetic mothers and received 24‐h nursery care. They were cared for and fed with infant Similac^®^ formula by mouth every 4–6 h, and placed in an incubator (36°C) for thermal support. They were monitored daily by veterinary staff and were euthanized on day of life 2–5 with 20 mg/kg of IV pentobarbital, followed by exsanguination. Liver samples were snap frozen at the time of necropsy.

#### Adult animals

Four healthy, nondiabetic adult female baboons maintained at the Southwest National Primate Research Center at TBRI (San Antonio, TX) were studied. The animals were fed an *ad libitum* chow diet supplemented with seeds and corn and were housed in outdoor group caging.

Based on our preliminary findings, preterm baboons have a relative FBPase gene expression of 2.3 ± 0.5 and term of 1.3 ± 0.6, with a 5% significance level and 80% power, and sample size of five animals per group was calculated.

All studies were approved by the Institutional Animal Care Committee at the University of Texas Health Science Center in San Antonio. Animal experiments were conducted in accordance with NIH‐ Public Health Service Policy on Humane Care and Use of Laboratory Animals. UTHSCSA is accredited with AAALAC, Int.

### Western blot analysis

Activation of hepatic gluconeogenic proteins was measured from extracted frozen liver tissues. Liver samples were homogenized in ice‐cold lysis buffer (containing 20 mmol/L Tris (pH 7.5), 10 mmol/L sodium pyrophosphate, 100 mmol/L sodium fluoride, 2 mmol/L sodium orthovanadate, 5 mmol/L EDTA (pH 8.0), 1% Nonidet P‐40, 1 mmol/L PMSF, 3 mmol/L benzamidine, 10 *μ*g/mL leupeptin, and 10 *μ*g/mL aprotinin). Homogenates were centrifuged and supernatants were used to measure protein concentrations with the NanoDrop ND‐1000 Spectrophotometer from Thermo Fisher Scientific Inc.^®^ (Waltham, MA). Lysate proteins were separated by 10% SDS‐PAGE and transferred to nitrocellulose membranes using reagents purchased from Bio‐Rad Laboratories, Inc. (Hercules, CA). Membranes were blocked with Tris‐buffered saline and 10% nonfat dry milk, then incubated overnight at 4°C with their respective primary antibody. Protein content was measured using primary antibodies against the following gluconeogenic enzymes: PEPCK‐Cytosolic (PEPCK‐C), PEPCK‐Mitochondrial (PEPCK‐M), G6Pase‐*α*, G6Pase‐*β*, FBPase (Santa Cruz Biotechnology, Santa Cruz, CA). Transcription factor FOXO1 and pFOXO1 (Ser256) were also measured (Santa Cruz Biotechnology). Bound antibodies were detected with ECL anti‐rabbit immunoglobulin‐horseradish peroxidase‐linked whole antibody (GE Healthcare UK Limited, Little Chalfont, UK) and by using Western Lighting Plus‐ECL reagents (Perkin Elmer, Waltham, MA). The intensities of the bands were quantified by densitometry using the imaging program ImageJ (National Institutes of Health, Bethesda, MD), with the results reported in arbitrary optical density (OD) units.

### PCR analysis

Gene expression levels were measured by extracting total RNA from frozen liver tissues using the RNeasy Mini Kit from Qiagen (Valencia, CA). Total RNA was reverse transcribed into cDNA using the High‐Capacity cDNA Reverse Transcription Kit from Life Technologies (Carlsbad, CA) according to the manufacturer's protocol. Relative quantitation of gene expression was accomplished with the TaqMan (Life Technologies, Foster City, CA) methodology using the relative standard curve method. TaqMan gene expression assays for PEPCK‐C (Hs00159918_m1), G6Pase‐*β* (Hs00292720_m1), FBPase (Hs00983323_m1), and FOXO1 (Hs01054576_m1) were obtained from Life Technologies. The quantity of the mRNA for each gene of interest was normalized to that of IPO8 (Hs00183533_m1). The real‐time quantitative PCR reactions were performed with 30–50 nanograms of total RNA converted to cDNA as the template. The PCR reactions consisted of the cDNA template, 1X Universal PCR Master Mix for Gene Expression (Life Technologies), and the gene‐specific assay mix. The PCR reactions were cycled in a 7900HT Sequence Detection System (Applied Biosystems, Foster City, CA). Data were analyzed using the SDS software (Applied Biosystems) and the amount of mRNA for each gene in the samples was determined by normalizing the mass of the target gene to the mass of the endogenous control gene.

### Statistical analysis

Statistical calculations and demographic distributions were performed with SPSS for Microsoft Windows^®^, (Version 17.0, SPSS, Inc., Chicago, IL). Differences between groups were determined utilizing ANOVA, followed by Bonferroni and Tukey test. Student's *t*‐test, Chi‐square, and Fisher's exact test were performed when appropriate. A *P *< 0.05 was considered to be statistically significant.

## Results

Demographics for the fetal, neonatal, and adult animals studied are shown in Table 1. Serum glucose levels were similar between the fetal animals and increased with postnatal life.

**Table 1.** Animal characteristics of baboons used to investigate developmental differences of key hepatic gluconeogenic molecules.

Gestational age	Gestation (% of term)	Female	Weight (g)^1^	Glucose (mg/dL)^1^
125d	67	2/5	412.7±13.7	40.6±2.1
140d	75	4/5	551.2±30.0	37.0±3.2
175d	95	1/5	970.8±62.0	31.4±2.1
Term	100	4/5	878.0±58.9	68.0±17.8
Adult	N/A	4/4	N/A	87.0±16.6^2^

d, day; g, grams.

^1^ Values are means ± SE.

^2^ Results are pooled from various adult controls.

### Gluconeogenic molecules during fetal development

#### Fetal PEPCK increases with advancing gestational age

**Figure 1.**
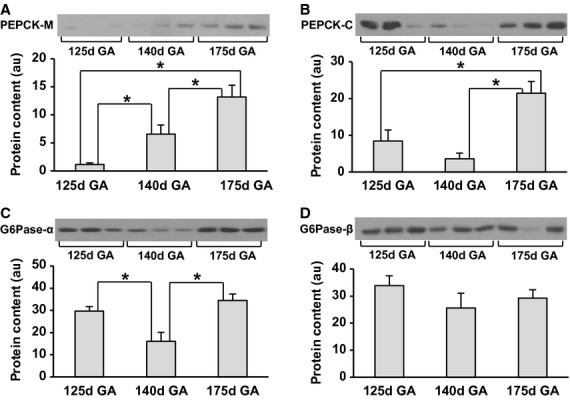
Effect of gestational age on protein content of key hepatic gluconeogenic molecules in fetal baboons. PEPCK‐M (A), PEPCK‐C (B), G6Pase‐*α* (C), G6Pase‐*β* (D) were measured by Western blotting. *n* = 5 per group. Representative blots from three animals per group are also shown. d = day and GA = gestational age. Data are means ± SE. *P < 0.01.

**Figure 2.**
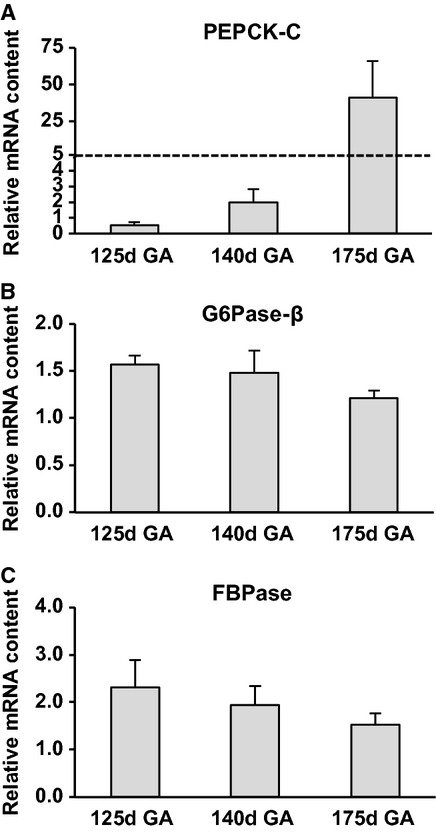
Effect of gestational age on gene expression of key hepatic gluconeogenic molecules in fetal baboons. PEPCK‐C (A), G6Pase‐*β* (B), FBPase (C) were measured by qrt‐PCR. Dotted line indicates split graph due to the high mRNA content of 175d GA animals relative to their counterparts. *n* = 5 per group. d = day and GA = gestational age. Data are means ± SE. *P < 0.01.

Protein content of fetal hepatic PEPCK‐M increased with advancing gestational age (Fig. 1A) with a 9.6‐fold increase from 125d GA to 175d GA (*P *< 0.001). PEPCK‐C protein content increased by 2.5‐fold from 125d GA to 175d GA (*P *< 0.05) (Fig. 1B). Similarly, mRNA levels of fetal hepatic PEPCK‐C showed a trend to increase from the most immature animals to the near‐term animals (Fig. 2A); however, it did not reach statistical significance.

#### Fetal G6Pase decreases transiently in late gestation

The protein content of fetal G6Pase‐*α* transiently decreased at 140d GA by 41% (*P* < 0.01) (Fig. 1C). G6Pase‐*β* protein and mRNA expression did not change during fetal life (Figs 1D, 2B).

#### Fetal FBPase does not change throughout gestation

In contrast to fetal PEPCK, mRNA expression of fetal hepatic FBPase was similar across all gestational ages (Fig. 2C).

#### Fetal FOXO1 remains unchanged throughout fetal life

**Figure 3.**
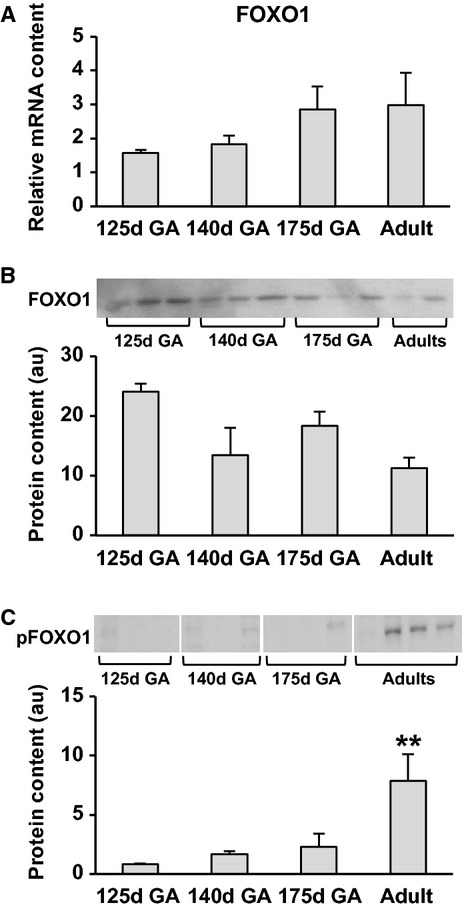
Developmental differences in protein content, gene expression and phosphorylation of FOX01. FOXO1 were measured by qrt‐PCR (A) and Western blotting (B and C) adults (*n* = 4) and neonates (*n* = 20);, *n* = 4–6 per group, representative blots are also shown. d = day and GA = gestational age. Data are means ± SE. *P < 0.05, ** *P *< 0.01 versus all groups.

The fetal protein content and gene expression of the hepatic transcription factor FOXO1 were similar across all gestational ages (Fig. 3A and B). Phosphorylated FOXO1 tended to increase with advancing gestation but it did not meet statistical significance (Fig. 3C).

### Gluconeogenic molecules during postnatal development

#### PEPCK increases with postnatal maturation

**Figure 4.**
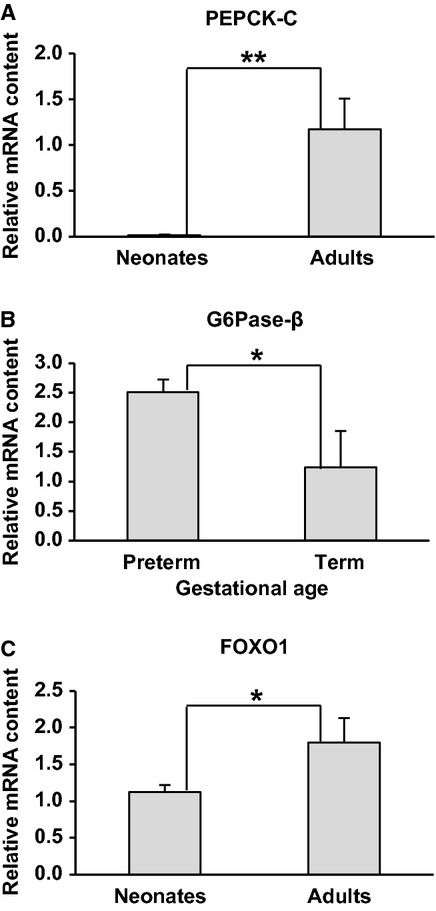
Postnatal changes in gene expression of key gluconeogenic molecules. PEPCK‐C (A), G6Pase‐*β* (B) and FOXO1 (C) were measured by qrt‐PCR. Adults (*n* = 4) and neonates (*n* = 20); preterm (*n* = 12), term (*n* = 6), d = day and GA = gestational age. Data are means ± SE. *P < 0.05 and **P < 0.001.

PEPCK‐C mRNA increased significantly from fetal life to adulthood (Fig. 4A); preterm baboons had <1% of the mRNA expression of adult baboons, and term animals had 2.5% mRNA of adult baboons (*P *< 0.001). However, PEPCK‐C and PEPCK‐M protein content was similar between fetal and postnatal animals.

#### Postnatal G6Pase decreases into adulthood

Preterm fetal animals had higher mRNA expression of G6Pase‐*β*, as compared to the term postnatal counterparts (*P *< 0.05) (Fig. 4B). G6Pase‐*α* and *β* subunit protein content was significantly higher in term newborn animals when compared to adult animals (*P *< 0.05) (data not shown).

#### FOXO1 gene expression and Phosphorylated FOXO1 increases into adulthood

FOXO1 gene expression in newborn baboons was 62% of adult baboons (Fig. 4C) when compared to all neonatal baboons regardless of gestational age (*P *< 0.05). While FOXO1 protein content was similar between fetal, neonatal, and adult baboons, phosphorylated FOXO1 in fetal baboons was only 10%, 20%, and 27% of adult baboons (125d, 140d, and 175d respectively, *P *< 0.001) (Fig. 3C).

#### FBPase protein content increases during postnatal life

Animals born at term gestation had a 2.2‐fold higher FBPase protein content than fetuses (*P *< 0.05), however, no differences were observed with maturation into adulthood (data not shown). FBPase mRNA expression was similar between preterm, term, and adult baboons (data not shown).

## Discussion

In this study, we found significant differences in the protein content and gene expression of hepatic gluconeogenic molecules throughout fetal and postnatal life. In particular, we demonstrated that phosphorylated FOXO1 was significantly reduced in the liver of preterm fetal baboons compared to adults. FOXO1 is a central transcription factor promoting the transcription of the gluconeogenic enzymes PEPCK and G6Pase (Cheng and White 2011). Insulin suppresses gluconeogenesis through phosphorylation of FOXO1, which promotes FOX01 cytoplasmic translocation and ubiquitination, inhibiting FOXO1‐mediated gene transcription (Cheng and White 2011; Kerecman et al. 2013). Therefore, a low phosphorylation of FOX01 in preterm infants may play a role in their high incidence of hyperglycemia; whether these differences persist under insulin‐stimulated conditions remains to be determined.

During early fetal development, we demonstrated that PEPCK, the first rate‐limiting step of the gluconeogenesis pathway (Girard 1986), had striking differences when compared to other gluconeogenic molecules during both fetal and postnatal development with upregulation of fetal PEPCK during late gestation suggesting increased gluconeogenesis in preparation for extra‐uterine life. After birth, gene expression of PEPCK was significantly higher in adult baboons compared to fetal and neonatal animals. The sequential upregulation of PEPCK indicate this enzyme may be critical to sustaining glucose production during late gestation and postnatal life as liver PEPCK was found recently to be strongly related to glycemia in humans (Li et al. 2013). Studies in rodents have suggested that the activation of hepatic PEPCK gene transcription at birth initiates gluconeogenesis (Benvenisty et al. 1985). The timing of this initiation is critical for sustaining postnatal life as a delay results in hypoglycemia; however, early abnormal upregulation of PEPCK may lead to neonatal hyperglycemia. Although gene expression of PEPCK was significantly higher in adult baboons, we found no differences in protein content of PEPCK in adults compared to fetal animals. These differences in protein and gene expression indicate that other modes of regulation may be taking place downstream of transcription, and further studies are required to elucidate these processes.

Previous studies have shown low expression of gluconeogenic enzymes in the rat liver until after birth concluding the induction of PEPCK‐C is largely postnatal (Ballard 1971; Benvenisty et al. 1985; Cassuto et al. 1999; Postic et al. 2004). In contrast, fetal lambs and goats have shown developmental changes in PEPCK expression before birth (Dhanotiya and Bhardwaj 1988; Sunehag et al. 1993). A potential confounder might be the fetal environment such as hypoglycemia, hypoinsulinemia, intrauterine growth, and/or nutrient restriction, which have been found to induce hepatic PEPCK‐C during late gestation (Philippidis et al. 1972; Torrecilla et al. 2012; Magnusson et al. 2014). In this study, we had no evidence of potential environmental confounders. Understanding the effects of environmental factors on the gluconeogenic pathway might help elucidate the persistent gluconeogenesis seen in preterm infants despite hyperglycemic conditions (Thorn et al. 2013).

Both mitochondrial and cytosolic PEPCK are likely key players for postnatal adaptation and, therefore, may contribute to developmental and long‐term dysfunction. At mid‐gestation, hepatocytes have few mitochondria (Jones and Rolph 1985), which may explain the lower levels of PEPCK‐M that were observed in the most immature animals. Contrary to other studies (Philippidis et al. 1972; Torrecilla et al. 2012; Wu et al. 2014) we found that both mitochondrial and cytosolic PEPCK changed with advancing maturation while fetal sheep have reported mixed results (Ballard and Oliver 1965; Edwards et al. 1975; Sunehag et al. 1993, 1993). The differences may be species‐specific PEPCK distribution, as rat and mouse species only have 10% PEPCK‐M, while humans have 50% PEPCK‐M (Hanson 2009). PEPCK abnormalities have been shown to have an important role in diabetic adults with hepatic insulin resistance (Stevenson et al. 1976; Van Kempen et al. 2003; Kumashiro et al. 2013). Therefore, it is important to determine the normal developmental gluconeogenic pathway in order to elucidate which specific factors and enzymes have the largest contribution to altered glucose regulation.

The mechanism of fetal hepatic PEPCK induction is still not clearly understood. FOXO1 is thought to bind as a transactivator to the promoters of PEPCK and G6Pase for stimulating gluconeogenic gene expression (Accili and Arden 2004). Insulin acts through a signaling cascade to phosphorylate FOXO1 and promote cytoplasmic translocation, which inhibits FOXO1‐mediated gene transcription (Guo 2014). Prior studies in fetal baboons delivered prematurely at 67%, 75%, and 94% gestation found that there were no differences in the content of hepatic insulin signaling proteins: Insulin receptor (IR)‐*β*, IR substrate‐1, p85 subunit of phosphatidylinositol 3‐kinase, and Akt (Blanco et al. 2010). Furthermore, preterm animals delivered at 67% gestation and full‐term animals had similar fasting insulin levels at birth (C. L. Blanco, unpubl. data). Notably, although phosphorylated (inactive) FOXO1 tended to increase with advancing gestation and significantly increased in adulthood, the gene expression of FOXO1 target gene PEPCK continued to increase while G6Pase did not. With increasing phosphorylated FOXO1, repressed mRNA expression of both PEPCK and G6Pase might be expected. However, PEPCK gene expression increased despite rising phosphorylated (inactive) FOXO1 in preterm baboons, which may indicate alternative pathways for glucose regulation in prematurity.

Developmental regulation of FOXO1 expression has been studied in rodents where mRNA expression was barely detectable at two‐thirds gestational age in hepatocytes and dramatically increased by 88% completed gestation (Stark et al. 2014). Modulation of FOXO1 with fetal exposure to nutrient restriction and hypoglycemia has been found in fetal sheep (Torrecilla et al. 2012). However, the role of FOXO1 has not been well studied in the naïve human fetus. The importance of understanding normal fetal development of FOXO1 is highlighted by the lack of data on interactions between FOXO1 and key gluconeogenic enzymes. In this nonhuman primate model, we studied transcription factors in healthy, unstressed fetal animals and we can potentially understand short and long‐term effects of perinatal environment.

In contrast to other studies, our data suggest that FBPase does not change during fetal development. In prior mammalian studies G6Pase and FBPase were sequentially upregulated at around 67% of gestation toward term gestation (Jones and Ashton 1976; Dhanotiya and Bhardwaj 1988; Fowden et al. 1991; Mitanchez 2007). Conversely, when we compared all of the fetal preterm animals to those born at term, we found higher G6Pase mRNA expression in the most immature fetuses. Although these differences may be species specific, many of the previous animal studies were done over 20 years ago, and the differences found on this study might also be due to advancements in technology and the use of more accurate techniques for analyzing gene expression. Another possibility is that we studied additional groups in late gestation and not a snap shot in mid‐late gestation. We found a transient decrease in G6Pase‐*α* at 75% completed gestation with subsequent upregulation after that point; if we had not studied the 67% completed gestation fetuses, our interpretation would have been different. Studies in fetal sheep found EGP did not start until near‐term gestation and was found only in fetuses with induced G6Pase (Fowden et al. 1998). Unfortunately, in this study we didn't evaluate EGP, but the basal glucose levels were similar between fetal groups (Table 1).

A limitation to this study is that premature fetal baboons have increased hepatic extramedullary hematopoiesis compared to term and adult animals (Kerecman et al. 2013). These histopathologic differences may contribute to the developmental differences in hepatic gluconeogenic molecules and requires further investigation. On the other hand, this will likely be the case in the human premature infants as they also have increased extramedullary hematopoiesis and therefore, this study is highly translational.

In summary, we found significant gestational differences in key gluconeogenic molecules. In particular, upregulation of phosphorylated FOXO1 was seen in adult livers compared to premature fetal/neonatal liver specimens. These findings suggest that decreased FOXO1 phosphorylation may play a role in the hepatic insulin resistance reported in prematurity. Additionally, hepatic PEPCK seems to have the largest changes during fetal development, and tissue‐specific modulation of PEPCK might be a potential therapeutic target for glucose regulation and hepatic insulin resistance.

Babies born preterm have a greater chance of developing insulin resistance, glucose intolerance, and high blood pressure when they become young adults (Hovi et al. 2007; Kajantie and Hovi 2013). Thus, understanding the fetal development of gluconeogenic enzymes will help us in the management of infants with aberrant glucose control, and provide a better understanding of glucose metabolism and the implications of perinatal factors in this vulnerable population.

## Acknowledgments

We thank the personnel from the Veterinarian Services at UTHSCSA, the Oklahoma Primate Center, and the Texas Biomedical Research Institute for their dedication and support for this project.

## Conflict of Interest

There is no conflict of interest that could be perceived as prejudicing the impartiality of the research reported.
